# P010 A Diagnostic Odyssey: Thoracic Outlet Syndrome and Clavicular Enlargement – What’s in the name?

**DOI:** 10.1093/rap/rkag113.040

**Published:** 2026-11-03

**Authors:** Roshni Patel, Oliver O’Sullivan, Muhammad Usman, Anwen Wright, Kulsum Khan, Ira Pande

**Affiliations:** Nottingham University Hospitals NHS Trust, Nottingham, United Kingdom; Nottingham University Hospitals NHS Trust, Nottingham, United Kingdom; Nottingham University Hospitals NHS Trust, Nottingham, United Kingdom; Nottingham University Hospitals NHS Trust, Nottingham, United Kingdom; Nottingham University Hospitals NHS Trust, Nottingham, United Kingdom; Nottingham University Hospitals NHS Trust, Nottingham, United Kingdom

## Abstract

**Introduction:**

Diagnostic labels can both guide and mislead clinical reasoning. We present a 53-year-old woman with a history of thoracic outlet syndrome who developed progressive clavicular enlargement over many years, raising concern for either malignant or inflammatory disease. Retrospective review of imaging performed eight years before the onset of enlargement revealed subtle clavicular bone oedema, indicating that pathology had been present long before it became clinically apparent. The diagnostic journey ultimately culminated in SAPHO syndrome (synovitis, acne, pustulosis, hyperostosis and osteitis), highlighting its overlap with psoriatic arthritis, the value of whole-body MRI, and the importance of multidisciplinary evaluation.

**Case description:**

A 53-year-old woman was referred with a four-year history of progressive right clavicular enlargement. She had been diagnosed with thoracic outlet syndrome 14 years earlier, presenting with episodic right arm pain and colour change; MRI demonstrated subclavian vein compression, and she was managed conservatively with good symptomatic improvement. Although the original report made no reference to bone pathology, retrospective review identified subtle right clavicular bone oedema predating overt enlargement by approximately eight years, reassuring clinicians that malignancy was unlikely and suggesting an inflammatory process had been evolving silently before diagnosis.

Over subsequent years she developed an enlarging, intermittently painful clavicular mass without overlying skin change. Inflammatory markers remained normal, but a mixed sclerotic lesion on radiography prompted rheumatology referral and multidisciplinary discussion. She also reported palmoplantar pustulosis, quiescent for three decades.

Examination revealed a hard, non-expansile swelling of the right clavicle and supraclavicular region, without active synovitis, nail disease or psoriasis. CT showed hyperostosis, sclerosis, periostitis and bony expansion involving the clavicle, sternoclavicular joints and manubrium. Whole-body MRI was subsequently performed to assess disease burden and identify further sites of activity, revealing extensive anterior chest-wall involvement together with multiple clinically silent thoracic vertebral lesions, including vertebral body and endplate oedema with subtle erosive change.

In the context of characteristic imaging and previous palmoplantar pustulosis, SAPHO syndrome was diagnosed. Adalimumab produced marked symptomatic improvement and reduced clavicular swelling. However, surveillance whole-body MRI later showed new subclinical vertebral and sacral lesions despite clinical remission. During follow-up, she disclosed psoriasis in her 20s and a recent solitary plantar lesion, raising the possibility that her axial symptoms reflected psoriatic disease in addition to SAPHO syndrome. She was referred to a national tertiary SAPHO centre, where PET imaging confirmed active spinal inflammation and excluded malignancy. Bisphosphonate therapy with zoledronic acid led to further symptomatic improvement.

**Discussion:**

This case highlights several diagnostic and therapeutic challenges inherent to SAPHO syndrome, particularly its overlap with other inflammatory musculoskeletal disorders. SAPHO has been described as an umbrella syndrome encompassing a broad spectrum of osteoarticular and dermatological phenotypes, contributing to substantial heterogeneity in presentation and management. The coexistence of historical palmoplantar pustulosis, subsequent psoriasis, anterior chest wall hyperostosis and axial inflammation blurred the distinction between SAPHO syndrome and psoriatic spondyloarthritis, raising the question of whether these conditions represent overlapping entities rather than discrete diagnoses.

A particularly striking feature was the retrospective identification of clavicular bone oedema on the initial MRI performed during investigation of thoracic outlet syndrome. This preceded clinically apparent clavicular enlargement by approximately eight years, suggesting inflammatory bone disease may have been evolving long before diagnosis became apparent and highlighting the prolonged diagnostic latency frequently observed in SAPHO syndrome.

Advanced imaging proved pivotal throughout the diagnostic journey. Whole-body MRI is not routinely employed in rheumatology practice but was selected to determine disease extent and identify additional sites of activity. It confirmed characteristic anterior chest wall involvement and demonstrated multiple clinically silent vertebral lesions, aligning with emerging evidence from large SAPHO cohorts showing abundant subclinical spinal lesions that may remain undetected without whole-body imaging. These findings substantially increased diagnostic confidence and informed treatment decisions.

The case also demonstrates the challenge of defining treatment response. The patient experienced near-complete symptomatic remission on adalimumab, with significant reduction in clavicular swelling and persistently normal inflammatory markers. However, surveillance imaging showed progression with new vertebral and sacral lesions. This discordance raised uncertainty regarding ongoing SAPHO activity, secondary anti-TNF failure, or an alternative process such as axial psoriatic disease. Multidisciplinary and tertiary-centre input was essential, with PET imaging excluding malignancy while confirming active spinal inflammation, guiding escalation to zoledronic acid, which produced further clinical improvement.

**Key learning points:**

• **Diagnostic complexity–**SAPHO syndrome remains difficult to diagnose because of its heterogeneous presentation and the variable timing between musculoskeletal and dermatological manifestations. Characteristic imaging features such as anterior chest-wall hyperostosis and sterile osteitis are often more informative than active skin disease, which may be absent, remote, or only recognised retrospectively.

• **Prolonged diagnostic latency—**Diagnostic delay can occur when early symptoms have plausible alternative explanations. In this case, subtle clavicular bone oedema seen on MRI for thoracic outlet syndrome many years earlier preceded clinically apparent enlargement by approximately eight years, suggesting inflammatory bone disease may evolve long before overt structural change.

• **Whole-body MRI value—**Whole-body MRI is particularly valuable in SAPHO syndrome. It confirmed the characteristic anterior chest-wall pattern and identified extensive, clinically silent vertebral lesions that would otherwise have remained undetected. Emerging evidence indicates subclinical spinal involvement is common and contributes significantly to disease burden.

• **Assessing treatment response—**Clinical improvement, normal inflammatory markers and patient-reported benefit do not necessarily equate to complete disease control. Despite marked symptomatic improvement on adalimumab, surveillance imaging revealed new vertebral and sacral lesions, highlighting discordance between clinical and radiological outcomes.

• **Diagnostic overlap—**Later emergence of psoriasis raised the possibility of axial psoriatic arthritis, prompting reconsideration of whether this represented SAPHO syndrome, psoriatic spondyloarthritis, or overlapping disease. Practical management implications were similar, with biologic therapy and bisphosphonate treatment appropriate in either scenario. This reflects the central question posed by this case: ***when diagnoses share pathophysiological and therapeutic pathways, how much is truly in the name?***

• **Multidisciplinary input—**Multidisciplinary review and specialist tertiary-centre input were essential in navigating diagnostic uncertainty, interpreting complex imaging findings and guiding ongoing management.

